# Molecular Basis and Ecological Relevance of *Caulobacter* Cell Filamentation in Freshwater Habitats

**DOI:** 10.1128/mBio.01557-19

**Published:** 2019-08-20

**Authors:** Kristina Heinrich, David J. Leslie, Michaela Morlock, Stefan Bertilsson, Kristina Jonas

**Affiliations:** a Science for Life Laboratory, Department of Molecular Biosciences, The Wenner-Gren Institute, Stockholm University, Stockholm, Sweden; b Science for Life Laboratory, Department of Ecology and Genetics, Uppsala University, Uppsala, Sweden; The Ohio State University School of Medicine

**Keywords:** *Caulobacter crescentus*, biofilms, cell cycle, cell shape, environmental signals, freshwater habitats, stationary phase

## Abstract

All living cells are characterized by certain cell shapes and sizes. Many bacteria can change these properties depending on the growth conditions. The underlying mechanisms and the ecological relevance of changing cell shape and size remain unclear in most cases. One bacterium that undergoes extensive shape-shifting in response to changing growth conditions is the freshwater bacterium Caulobacter crescentus. When incubated for an extended time in stationary phase, a subpopulation of C. crescentus forms viable filamentous cells with a helical shape. Here, we demonstrated that this stationary-phase-induced filamentation results from downregulation of most critical cell cycle regulators and a consequent block of DNA replication and cell division while cell growth and metabolism continue. Our data indicate that this response is triggered by a combination of three stresses caused by prolonged growth in complex medium, namely, the depletion of phosphate, alkaline pH, and an excess of ammonium. We found that these conditions are experienced in the summer months during algal blooms near the surface in freshwater lakes, a natural habitat of C. crescentus, suggesting that filamentous growth is a common response of C. crescentus to its environment. Finally, we demonstrate that when grown in a biofilm, the filamentous cells can reach beyond the surface of the biofilm and potentially access nutrients or release progeny. Altogether, our work highlights the ability of bacteria to alter their morphology and suggests how this behavior might enable adaptation to changing environments.

## Abstract (precis)

Many bacteria drastically change their cell size and morphology in response to changing environmental conditions. Here, we demonstrate that the freshwater bacterium Caulobacter crescentus and related species transform into filamentous cells in response to conditions that commonly occur in their natural habitat as a result of algal blooms during the warm summer months. These filamentous cells may be better able to scavenge nutrients when they grow in biofilms and to escape from protist predation during planktonic growth. Our findings suggest that seasonal changes and variations in the microbial composition of the natural habitat can have profound impact on the cell biology of individual organisms. Furthermore, our work highlights that bacteria exist in morphological and physiological states in nature that can strongly differ from those commonly studied in the laboratory.

## INTRODUCTION

Bacterial cells are usually associated with a characteristic morphology and size. While most common laboratory bacteria are characterized by their rod and coccus shapes, in nature an abundance of other morphologies have been observed, ranging from the branched filaments of *Streptomyces* to the helically coiled shapes of spirochetes (1). These species-specific morphologies are genetically encoded and presumably confer a competitive advantage to justify their maintenance. However, despite their characteristic morphologies, many bacteria can drastically change their appearance in response to changing environmental conditions. This form of morphological variation is reversible and occurs without genetic changes. A number of distinct mechanisms that operate in response to a variety of different environmental triggers have been described previously (2, 3). Such mechanisms likely help cells survive adverse conditions; however, experimental evidence demonstrating how cells benefit from morphological changes is in most cases absent.

In uropathogenic Escherichia coli in a mouse bladder infection model, filamentation occurs as a result of induction of the SOS response, which activates the expression of *sulA*, encoding an inhibitor of cell division. The SOS-induced block of cell division leads to cell elongation, which was shown previously to protect cells from phagocytosis (4). Filamentous bacteria have also been observed frequently in freshwater ecosystems, where their abundance was low compared to small bacteria but their contribution to the biovolume surprisingly high, due to their increased size (5, 6). However, such complex communities and environments are difficult to study and little is known about the role bacterial filamentation might play.

The alphaproteobacterium Caulobacter crescentus is a well-established model organism found in freshwater habitats, where it lives both planktonically and in biofilms (7, 8). Freshwater lakes and rivers experience great fluctuations in nutrient availability and microbial composition throughout the year, requiring organisms to be able to adapt and respond to ensure their survival. In addition, biofilm-forming organisms need to be able to adapt to the conditions within the biofilm, which often differ greatly from those of the surrounding milieu (9–11).

It has previously been shown that C. crescentus undergoes morphological changes in response to external cues (12, 13). Similarly to E. coli, DNA damage in C. crescentus activates the expression of small inhibitory proteins that directly interfere with the cell division machinery, causing cells to become elongated (13, 14). More recent work from our group showed that salt and ethanol stresses lead to cell filamentation. In these cases, however, filamentation was due to the rapid dephosphorylation and destabilization of the cell cycle master regulator CtrA, an essential transcriptional regulator of cell division and cell cycle genes (12).

Another conspicuous morphological transition has been observed in the late stationary phase, during which some cells morph into long, helical filaments (15, 16). Previous work showed that these filaments make up only a small subpopulation of the culture that represents the viable fraction of cells. The vast majority of small cells were found to be dead. The helical shape of these filaments is mediated by crescentin (CreS), an intermediate filament-like protein required for the vibrioid shape of C. crescentus (17). It was also suggested previously that these filaments are more resistant to some stress conditions than early-stationary-phase cells (15). However, their low abundance in the culture prevented any detailed molecular studies of these cells without interference from the nonfilamentous fraction.

Here, we developed an approach to isolate filamentous cells that enables a detailed molecular analysis of their physiology. Cell biological assays in combination with proteomics approaches revealed that most of the critical cell cycle regulators are absent, leading to a shutdown of the critical cell cycle processes of DNA replication and cell division while growth and metabolism continue, albeit slowly. We found that the combination of low levels of phosphate, alkaline pH, and high levels of ammonium leads to this phenotype and that such conditions also occur in the native environment of *Caulobacter* as a consequence of algal bloom. Finally, our data suggest that when grown in biofilms, a filamentous morphology may help cells to access more nutrient-rich surroundings.

## RESULTS

### Prolonged growth in complex medium leads to formation of a subpopulation of filamentous cells that can be purified by density gradient centrifugation.

**FIG 1:**
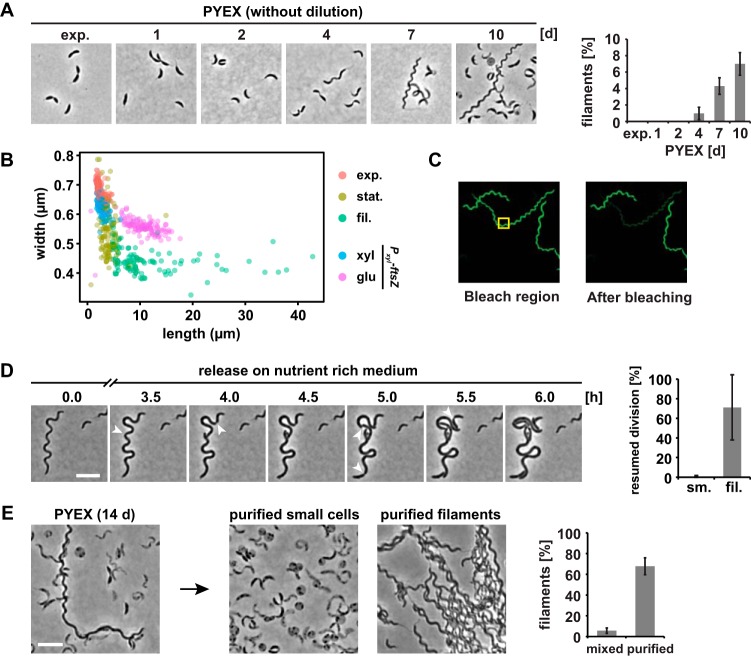
Prolonged growth in complex medium leads to formation of a subpopulation of filamentous cells that can be purified by density gradient centrifugation. (A) Phase-contrast microscopy pictures and quantification of numbers of filamentous cells of C. crescentus NA1000 in PYEX over 10 days (d). The quantification is based on results from at least three independent experiments. exp., exponential-phase growth. (B) Length and width of *Caulobacter* cells during exponential-phase growth (exp; OD_600_, <0.4) and early (24-h)-stationary-phase growth (stat.) in PYEX, of purified filamentous cells from a 10-day-old PYEX culture (fil.), and of *P_xyl_-ftsZ* cells grown for 3 h with xylose (xyl) or glucose (glu) to deplete FtsZ. A total of 100 cells from each condition are shown. For the purified filamentous cells, cells more than twice the mean length of the exponential-phase cells were included in the analysis. (C) Diffusion of a fluorescent protein within a filamentous cell. The area marked with a yellow box was repeatedly bleached. See Materials and Methods for details. (D) Recovery time-lapse images of cells from a 10-day-old PYEX culture grown on agarose pads containing fresh PYE medium. Arrowheads indicate invaginations at future division sites. The quantification bar graph at the right shows means and standard deviations of results from two independent experiments (see also Movie S1). Bar, 5 μm. (E) Phase-contrast pictures of a late-stationary-phase culture before and after density gradient centrifugation. The quantification shows percentages of filamentous cells before and after purification. Data represent means and standard deviations of results from at least two independent experiments. Bar, 5 μm.

Prolonged growth of wild-type Caulobacter crescentus in complex peptone-yeast extract (PYE) growth medium leads to the formation of a subpopulation of helical filamentous cells (Fig. 1A) (15). We found that filamentation was particularly pronounced during incubation in PYE medium supplemented with xylose. Under those conditions, filaments were first observed after 4 days and gradually increased in frequency, accounting for 7% of all cells observed after 10 days (Fig. 1A). In 10-day-old cultures, most filamentous cells are around five times longer than exponentially growing cells; however, several filaments reach around 20 times the length of exponential-phase cells (Fig. 1B). At the same time, they are also narrower than exponential-phase cells or FtsZ-depleted cells of similar length. Photobleaching experiments showed that the filamentous cell shape is a result of elongation of a single cell with one connected cytoplasm rather than chaining of connected but internally separated cells (Fig. 1C). Furthermore, time-lapse microscopy revealed that most filamentous cells were able to restart cell division when transferred to agarose pads supplemented with fresh PYE medium following an initial phase of cell volume increase (Fig. 1D; see also Movie S1 in the supplemental material). The majority of small cells from the late stationary phase were not able to resume growth and cell division following release to fresh medium (Fig. 1D), which is consistent with the notion that most of these cells are dead (15).

10.1128/mBio.01557-19.8 MOVIE S1 Recovery time-lapse movie of cells from late stationary phase. Cells from a 10-day-old PYEX culture were mounted on rich (PYE) medium agarose pads (1% agarose) and incubated at 30°C while being examined under a microscope. Download Movie S1, AVI file, 0.7 MB. Copyright © 2019 Heinrich et al. 2019 Heinrich et al. This content is distributed under the terms of the Creative Commons Attribution 4.0 International license.

Wortinger et al. reported previously that stationary-phase-induced filamentous growth of C. crescentus cannot be observed in M2G minimal medium (15), in which glucose is the sole carbon source. We observed that in M2G medium the culture pH drops to below 5 following entry into stationary phase and that the change in pH is accompanied by a strong reduction of cell viability (see Fig. S1A in the supplemental material). When we maintained the pH of M2G at 7 by adding NaOH as required, viability was increased, but filamentation was not observed (Fig. S1B). Switching the carbon source from glucose to an amino acid, for example, alanine or glutamate, raised the pH to 8 to 9 and led to cell filamentation (Fig. S1C), indicating that the metabolism of amino acids might play a role for filamentation in the late stationary phase.

10.1128/mBio.01557-19.1 FIG S1 Filament formation varies in different media. (A) Microscopy images of NA1000 after 10 days in PYEX or M2G with the corresponding pH values and spot assays of the cultures over time. Samples were taken and spotted on PYE plates. (B) Microscopy pictures and spot assays of NA1000 in M2G where the pH was kept at 7 by adding NaOH when required. (C) Phase-contrast microscopy pictures of NA1000 in minimal medium that contained glutamate or alanine as the carbon source. The pH of the culture is displayed in the images. Download FIG S1, PDF file, 0.4 MB. Copyright © 2019 Heinrich et al. 2019 Heinrich et al. This content is distributed under the terms of the Creative Commons Attribution 4.0 International license.

The filamentous cells comprise only a fraction of all cells in the late stationary phase. Thus, until now it was difficult to analyze these cells in isolation on the molecular level. We found that density gradient centrifugation performed using Percoll enabled the separation of the filamentous cells from the smaller cells and debris found in late-stationary-phase batch cultures. Using this approach, we were able to increase the proportion of filamentous cells from 7% to 70% (Fig. 1E).

### Uncoupling of cell growth from major cell cycle processes leads to cell filamentation.

**FIG 2:**
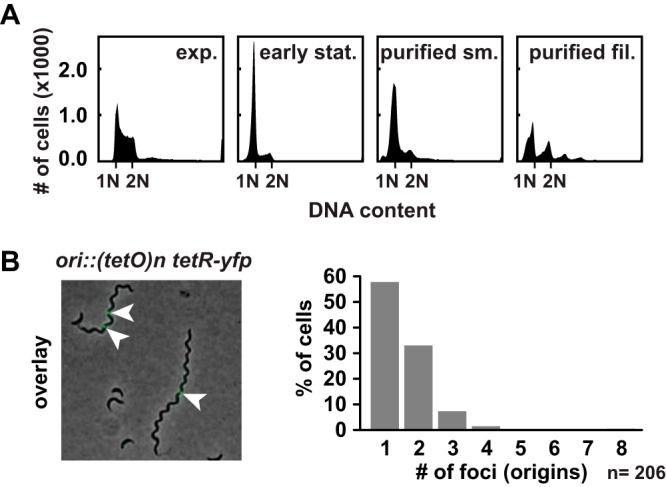
Late-stationary-phase cell filaments remain with mainly one or two chromosomes. (A) Flow cytometry profiles of cells from exponential-phase growth and early-stationary-phase growth in PYEX and small cells and filamentous cells after purification stained with SYTOX Green to measure the DNA content. Indicated are the fluorescent intensities corresponding to one chromosome (1N) and two chromosomes (2N). (B) Overlay of phase-contrast and fluorescence microscopy images of strain MT16 carrying a (*tetO*)_n_ array near the origin of replication fluorescently labeled with TetR-YFP after 10 days in PYEX. Arrowheads indicate fluorescently labeled origins. Quantification data at left show the number of foci in 206 filamentous cells.

Our method to enrich filamentous cells enabled us to investigate the molecular basis of filament formation. Bacteria blocked in cell division often accumulate multiple chromosomes (12, 18). Using flow cytometry, we did not observe chromosome accumulation in the filamentous cells isolated from the late stationary phase. The majority of cells (73%) contained only one or two fully replicated chromosomes, while only 19% of cells harbored three or four chromosomes, demonstrating that the number of chromosomes had not increased proportionally to cell length (Fig. 2A). Consistent with the flow cytometry data, we observed only a single focus or two foci in the majority (91%) of filamentous cells by the use of a fluorescent reporter operator system (FROS) that fluorescently labels the origin of replication (Fig. 2B). These data indicate a block of replication initiation additionally to the block of cell division, demonstrating that cell growth and metabolism are uncoupled from the major cell cycle processes of DNA replication and cell division in these cells.

**FIG 3:**
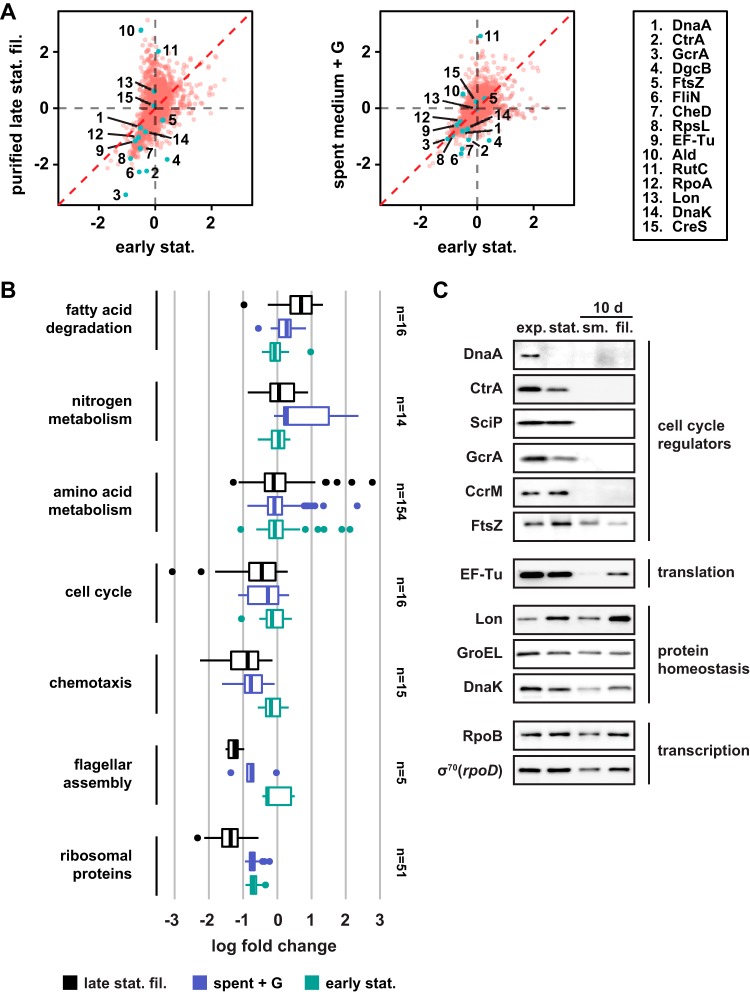
Critical cell cycle factors are downregulated in helical filamentous cells. (A) Proteome changes (shown as log_2_ ratios compared to exponential-phase cells) in late-stationary-phase filaments (left panel) and spent medium-induced filaments (right panel) compared to proteome changes in early-stationary-phase growth as obtained by quantitative mass spectrometry analysis. To generate spent medium-induced filaments, exponentially growing cells were resuspended in spent medium supplemented with glucose (G) for 24 h. (B) Average log_2_ fold change in protein levels compared to exponentially growing cells across proteins involved in selected processes (see also Table S1). The number of proteins in each category is indicated. (C) Western blot analysis of critical cell cycle regulators, proteins involved in transcription and translation, and protein homeostasis in cells from exponential-phase cells (exp.) and early-stationary-phase cells (stat.), and small (sm., 10 d) and filamentous (fil., 10 d) late-stationary-phase cells after purification.

To determine the molecular mechanisms responsible for the cell cycle arrest, we compared the proteomes of filamentous cells from late-stationary-phase and early-stationary-phase cells (24 h) to the proteomes of normally sized exponential-phase cells by quantitative proteomics using a tandem mass tag (TMT) isobaric labeling approach. Both conditions led to large changes in the proteome (Fig. 3A, left). Among the most strongly downregulated proteins in the filamentous cells compared to the exponential-phase cells were the master cell cycle regulators CtrA and GcrA, along with CtrA-dependent proteins such as flagellar and chemotaxis proteins (Fig. 3A and B). Notably, this downregulation was much more pronounced in the filamentous cells than in the early-stationary-phase cells. The replication initiator DnaA was also downregulated in the filamentous cells; however, the extent of the downregulation was similar to that seen in early-stationary-phase cells (Fig. 3A). In addition to proteins required for cell cycle progression and morphogenesis, ribosomal proteins were strongly downregulated in filamentous cells compared to both exponential-phase cells and early-stationary-phase cells (Fig. 3B), suggesting that although protein synthesis takes place in filamentous cells, its rate is significantly reduced. Proteins that were strongly upregulated in filamentous cells compared to exponential-phase cells included mostly metabolic proteins, for example, proteins required for amino acid metabolism (Fig. 3B). Of note, we did not observe an increased abundance of CreS or of other cytoskeletal proteins that could potentially account for the helical shape of the filamentous cells.

10.1128/mBio.01557-19.6 TABLE S1 Proteomics data. Download Table S1, XLSX file, 0.3 MB. Copyright © 2019 Heinrich et al. 2019 Heinrich et al. This content is distributed under the terms of the Creative Commons Attribution 4.0 International license.

We also measured the levels of selected proteins by Western blotting (Fig. 3C). These data confirmed that the master cell cycle regulators DnaA, CtrA, GcrA, CcrM, and SciP were eliminated and that the levels of the division protein FtsZ and the translation factor EF-Tu were downregulated in the filamentous cells. On the other hand, housekeeping proteins involved in transcription and protein homeostasis remained. Taken together, our data demonstrate that in filamentous cells, the cell cycle is arrested at the points of cell division and DNA replication initiation due to strong downregulation of the major cell cycle regulators DnaA, CtrA, CcrM, and GcrA. At the same time, the increasing length of the filaments over time suggests that cell growth and metabolism continue, at least at a low rate, in these cells.

### The combination of phosphate depletion, increased pH, and excess ammonium triggers the formation of helical filamentous cells.

“Stationary phase” is a term widely used to describe the cessation of growth in batch culture, but the specific stresses that cells experience during this phase are less well defined. Several stress conditions occur simultaneously, including the depletion of nutrients and the accumulation of metabolic products or changes in pH. We wondered what the nature of the stress signal is that triggers filamentous growth of C. crescentus in stationary phase.

**FIG 4:**
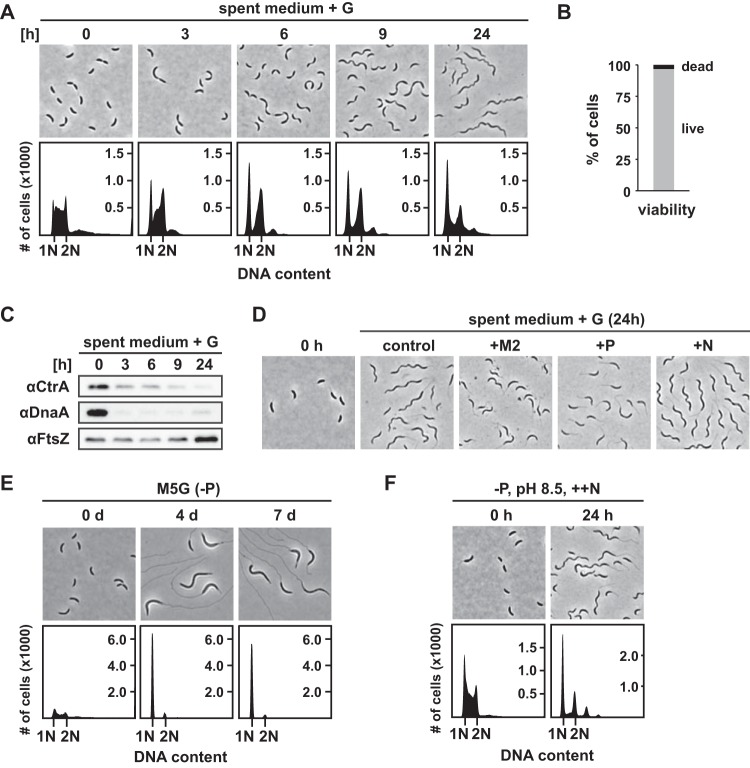
The combination of phosphate depletion, increased pH, and excess ammonium triggers the formation of helical filamentous cells. (A) Phase-contrast microscopy and flow cytometry profiles of NA1000 in spent medium supplemented with glucose taken at the times indicated. (B) Quantification of Live/Dead staining of NA1000 after 24 h in spent medium supplemented with glucose. (C) Western blot analysis of the samples shown in panel A. (D) Phase-contrast microscopy of NA1000 after 24 h in spent medium supplemented with glucose without further additional nutrients (control), with added M2 salts (M2), with added phosphate buffer (P), or with added ammonium (N). (E) Microscopy images and flow cytometry profiles of NA1000 in M5G minimal medium lacking phosphate over 7 days. The OD_600_ of the culture was kept below 0.4, and pH was adjusted to 7 with NaOH when required. (F) Phase-contrast images and flow cytometry profiles of NA1000 in minimal medium lacking phosphate, at pH 8.5, and with excess ammonium.

To tackle this question, we prepared “spent medium” by centrifuging and filtering the supernatant of stationary-phase cultures after 10 days of incubation and resuspended exponentially growing cells in this medium supplemented with glucose. Interestingly, we observed that nearly all exponential-phase cells transformed into filamentous cells with mainly one or two chromosomes (Fig. 4A) within 24 h of incubation, and Live/Dead staining showed that 97% of the cells were viable (Fig. 4B). Like the filamentous cells isolated from the late stationary phase, these filaments were thinner and had a helical shape. Moreover, the major cell cycle regulators, including CtrA and DnaA, as well as proteins involved in chemotaxis and motility were strongly downregulated during growth in spent medium (Fig. 3A and B; see also Fig. 4C). However, FtsZ, ribosomal proteins, and proteins involved in amino acid metabolism were less strongly downregulated, with levels that resembled those seen in early-stationary-phase cells (Fig. 3A and B; see also Fig. 4C). This might be explained by the fact that we supplemented the spent medium with glucose, which was not required for filament formation but which accelerated their emergence and increased the proportion of filamentous cells (Fig. S2). The higher rate of cell elongation potentially correlates with increased metabolic activity compared to the late-stationary-phase filamentous cells.

10.1128/mBio.01557-19.2 FIG S2 Glucose is not required for filament formation in spent medium. (A) Overlay of phase-contrast and fluorescent microscopy pictures of NA1000 growing in spent medium without glucose. Live/Dead staining was performed to visualize dead cells (red) and living cells (green). (B) Quantification of filament formation in samples from the experiment described in the panel A legend. (C) Quantification of viability of cells (stained as described in the panel A legend) by Live/Dead staining. Download FIG S2, PDF file, 0.2 MB. Copyright © 2019 Heinrich et al. 2019 Heinrich et al. This content is distributed under the terms of the Creative Commons Attribution 4.0 International license.

Next, we sought to determine if depletion of one of the main nutrients in the spent medium might have been the cause of the observed phenotypic response. Since addition of glucose to the spent medium still led to filamentation, we ruled out the possibility that exhaustion of carbon sources had triggered filamentous growth in the spent medium. To test whether depletion of phosphate or nitrogen was the cause of this cellular response, we supplemented the spent medium with phosphate or ammonium in concentrations corresponding to their final concentrations in M2G medium (Fig. 4D). Remarkably, addition of phosphate completely abolished filament formation. In contrast, addition of ammonium did not prevent filament formation; rather, it seemed to promote this response.

To test whether phosphate depletion alone is sufficient to trigger the formation of helical filaments, we monitored the morphology and replication status of cells in minimal medium lacking phosphate. Consistent with previous reports (19, 20), prolonged phosphate starvation led to cell elongation and an arrest of DNA replication (Fig. 4E), which was also observed in a Δ*phoB* strain (Fig. S3A), suggesting that these responses are PhoB independent. Furthermore, the Δ*phoB* strain also showed the same morphological response in late stationary phase as the wild type (Fig. S3B). In contrast to the filamentous cells that occurred in spent medium or late stationary phase, the phosphate-starved cells did not show a helical shape and also did not become thinner to the same extent (Fig. S3C). Thus, phosphate depletion does not seem to be the only cause of the morphological changes of C. crescentus in the late stationary phase.

10.1128/mBio.01557-19.3 FIG S3 Phosphate starvation in combination with high pH and ammonium induces the phenotype observed in late stationary phase, which is *phoB* independent. (A) Phase-contrast images and flow cytometry profiles of Δ*phoB* cells grown in M2G, then transferred to M5G without phosphate, directly after transfer and after 4 days. (B) Phase-contrast images of NA1000 and Δ*phoB* cells during exponential growth and after 10 days in PYEX. (C) Length and width of NA1000 in minimal medium under conditions of phosphate starvation, shown alongside the measurements of exponential-phase, early-stationary-phase, and late-stationary-phase cells from Fig. 1B for comparison. (D) Phase-contrast images and flow cytometry profiles of NA1000 in minimal medium under conditions of phosphate starvation (−P) or high pH (pH 8.5) or excess of ammonium (++N) after the times indicated. (E) Microscopy images and flow cytometry profiles of NA1000 in minimal medium treated with the combination of the stresses used as described for panel A. (F) Western blot analysis of CtrA and DnaA in cells subjected to all tested stresses in minimal medium over time. (G) Western blot analysis of the stresses phosphate starvation (−P), phosphate starvation and high pH (−P, pH 8.5), and phosphate starvation and excess ammonium (−P, ++N) after 2 and 4 days. Download FIG S3, PDF file, 0.7 MB. Copyright © 2019 Heinrich et al. 2019 Heinrich et al. This content is distributed under the terms of the Creative Commons Attribution 4.0 International license.

It is well established that batch culture growth on amino acids leads to increased pH and the secretion of ammonia into the medium (21, 22). Consistently, the spent medium from our stationary-phase culture had an alkaline pH of 8.5. We hypothesized that phosphate depletion in combination with increased pH and ammonium may lead to the characteristic helical filamentous cell shape observed in stationary phase. To test this idea, we prepared minimal medium formulated to have these three properties and analyzed the morphology and DNA replication status of cells incubated in this artificial medium. Indeed, incubation for 24 h resulted in the formation of filaments whose morphology resembled that of the filamentous cells from the late stationary phase (Fig. 4F). By testing the three conditions individually and in pairs, we confirmed that all three of these conditions were necessary for induction of the formation of helical filamentous cells that resemble those in spent medium and in the late stationary phase in terms of morphology and chromosome content (Fig. S3).

Altogether, our data suggest that the formation of helical filamentous cells in the late stationary phase is triggered by phosphate depletion in combination with increased pH 8.5 and excess of ammonium.

### C. crescentus is subjected to phosphate depletion in combination with alkaline pH and excess ammonium in its natural environment during persistent algal blooms.

**FIG 5:**
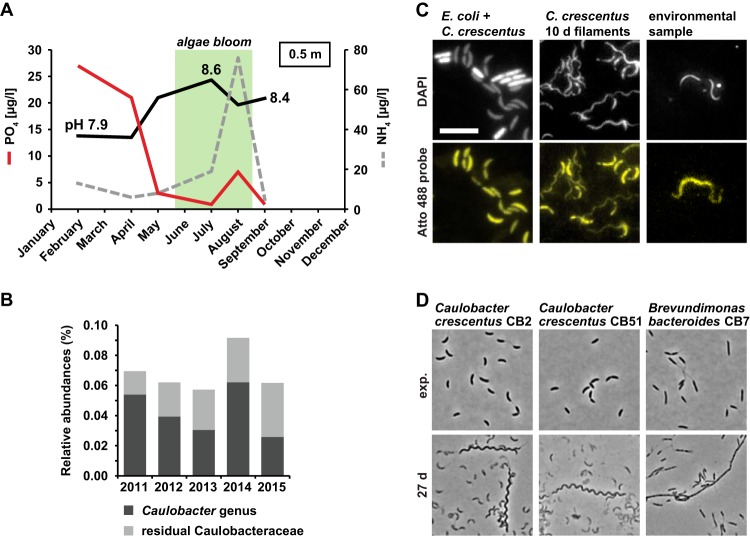
C. crescentus is subjected to phosphate depletion in combination with pH 8.5 and excess ammonium in its natural environment during algal bloom season. (A) Graph of phosphate and ammonium concentrations and pH of Lake Ekoln in Sweden taken from 0.5 m depth in 2017. Data were extracted from the national data host for lakes and watercourses (Miljödata MVM, SLU, Sweden)., A typical time frame for the occurrence of algal blooms is indicated in green. (B) Yearly average of the relative abundances of species belonging to the *Caulobacter* genus and residual Caulobacteraceae. (C) Fluorescence microscopy images of FISH analysis using a probe binding to organisms of the *Caulobacter* genus (see Fig. S4B). DAPI stain was used to visualize cells. Atto 488 probe signal indicates cells that were bound by the probe. Bar, 5 μm. (D) Phase-contrast microscopy images of C. crescentus CB2 and CB51 and Brevundimonas bacteroides CB7 during the exponential-growth phase and after 27 days of growth in PYEX.

*Caulobacter* and related species are commonly found in freshwater lakes and, under the conditions described above, which induced the formation of helical filamentous cells, frequently occur in surface waters of lakes during algal blooms. For example, in well-studied eutrophic Lake Ekoln (Sweden), phosphate concentrations drop sharply between April and May, while ammonium concentrations increase during the summer months in parallel to the development of an algal bloom (Fig. 5A). Because of high photosynthetic activity, pH also increases from 7.9 to 8.6 during this period. These conditions indeed match the experimental conditions for expected filamentation of *Caulobacter*, and similar seasonality in water chemistry is also seen in many other productive lakes, including the well-investigated microbial observatory of Lake Erken (23) (Fig. S4A).

10.1128/mBio.01557-19.4 FIG S4 Summertime phosphate depletion is a common feature in productive lakes. (A) Graph of phosphate and ammonium concentration and pH based on continuous sampling from Lake Erken in the years 2017 and 2018. The day of collection of an additional water sample for fluorescence *in situ* hybridization (FISH) analysis is indicated in blue. A typical time frame of the occurrence of algal blooms is indicated in green. (B) Alignment of the FISH probe sequence used in this study to different members of the Caulobacteraceae and E. coli. The arrow indicates the verified probe sequence (accession no. pB-377) used to design the new probe. (C) Additional examples of cells detected by FISH (see Fig. 5C). Download FIG S4, PDF file, 0.6 MB. Copyright © 2019 Heinrich et al. 2019 Heinrich et al. This content is distributed under the terms of the Creative Commons Attribution 4.0 International license.

To investigate possible filamentation of *Caulobacter* in its natural habitat, we decided to perform fluorescent *in situ* hybridization (FISH) on environmental samples collected from Lake Erken. Ongoing monitoring of bacterial communities in this lake with amplicon-based 16S rRNA analysis showed that *Caulobacter* species are persistently found in the upper water column over periods spanning several years (Fig. 5B). We collected water samples in late August 2018 from the upper water column and probed for *Caulobacter* cells using a specific Atto 488-labeled probe (Fig. S4B). Indeed, among the cells in the sample that were detected by staining with DAPI (4′,6-diamidino-2-phenylindole), we found 12 cells that showed a specific Atto 488 signal (corresponding to approximately 0.001% to 0.01%). Ten of the Atto 488-stained cells were filamentous and were comparable in size to filamentous C. crescentus cells; however, they did not exhibit the distinct helical shape of the filaments isolated from the late stationary phase (Fig. 5C; see also Fig. S4C). This phenotypic deviation might be attributable to the fact that not all three conditions were present at the time of the sampling; while the phosphate concentration was low (3.53 μg/liter) and the pH was elevated (8.3), the ammonium concentration (8.08 μg/liter) was still comparatively low. Phosphate starvation alone or in combination with increased pH leads to formation of nonhelical filamentous cells of C. crescentus (Fig. 4E; see also Fig. S3). Alternatively, the filamentous cells that we detected did not correspond to C. crescentus but rather to closely related *Caulobacter* species, for example, Caulobacter segnis and Caulobacter henricii, which our FISH probe is also expected to capture. These related species are likely to show different morphological characteristics during filamentation. Indeed, when we tested if other C. crescentus isolates and other members of the Caulobacteraceae would form filaments under conditions of growth to the late stationary phase, we observed that C. crescentus isolates CB2 and CB51 formed filaments resembling the filaments of our laboratory strain CB15N and that the more distantly related and nonvibrioid Brevundimonas bacteroides CB7 strain formed filamentous cells with a straight shape (Fig. 5D). These data show that the filamentous growth that we describe is not restricted to CB15-derived laboratory strains but also applies to other C. crescentus isolates and even other members of the Caulobacteraceae. This finding is consistent with a previous study whose results suggested that cell filamentation is a common response in aging cultures of members of the Caulobacteraceae (16).

In conclusion, the conditions that lead to the formation of helical filamentous cells occur in the natural habitat of C. crescentus during algal bloom and filamentous cells that likely belong to the genus *Caulobacter* can be observed at least during summer in the upper water column.

### Filaments can reach beyond the surface of the biofilm.

So far, our data had uncovered the molecular basis and the environmental triggers for filamentous growth of *Caulobacter*. We wondered if this form of filamentous growth might confer any advantage to the cells and might thus constitute an adaptive response.

Previously, it was suggested that the filamentous cells in late-stationary-phase cultures show increased resistance to different environmental stresses (15). With the ability to purify these cells from their culture, we analyzed their resistance to stress compared to that of exponential-phase and early-stationary-phase cells. We tested the resistance of the cells to oxidative stress, acid stress, sucrose stress, and heat stress. The stress resistance of filamentous cells was different from that of exponentially growing cells in most cases, being lower in the case of heat and acid stress and higher in the case of oxidative stress and sucrose stress (Fig. S5). Importantly, however, the filamentous cells from the late stationary phase were not markedly more resistant to any of the tested stresses than small cells from the early stationary phase.

10.1128/mBio.01557-19.5 FIG S5 Stress resistance of filamentous cells from late stationary phase compared to exponential-phase and early-stationary-phase cells. CFUs were counted before and after treatment under the corresponding stress condition, and the ratio was calculated to determine survival. exp, exponential-phase cells; stat, early-stationary-phase cells (24 h); fil, 10 d: filamentous stationary-phase cells from 10-day-old cultures. Error bars represent standard error of the means of results from at least three independent experiments, with the exception of the experiment testing sucrose sensitivity, which was performed twice. Significance was tested using Welch’s *t* test, with a significance threshold of *P ≤ *0.05. Download FIG S5, PDF file, 0.1 MB. Copyright © 2019 Heinrich et al. 2019 Heinrich et al. This content is distributed under the terms of the Creative Commons Attribution 4.0 International license.

**FIG 6:**
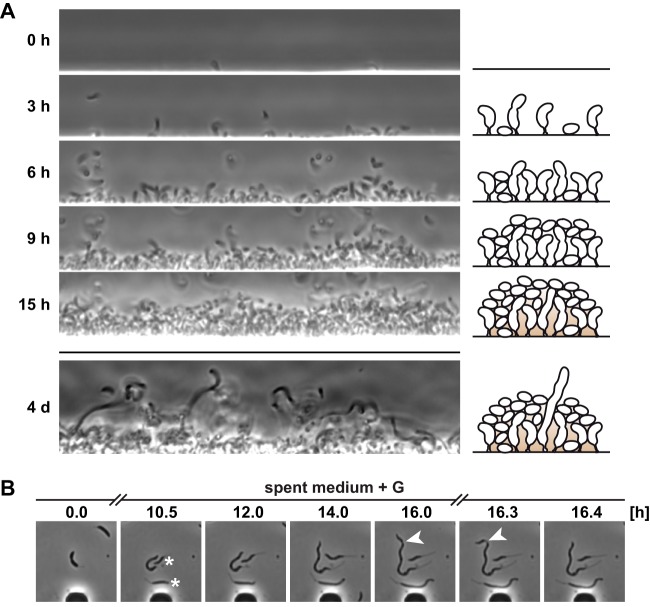
Filamentous cells are able to reach beyond the surface of a biofilm. (A) CB15 (holdfast^+^) cells grown in PYE overnight in a microfluidic device. The chamber was then flushed daily for several hours with diluted (1:10) PYE to wash away unattached cells and ensure a clear view of the biofilm. The upper panels show the colonization of a surface (see also Movie S2), and the lower panel shows filamentous cells extending from the biofilm after 4 days (see also Movie S3). A schematic sketch of the colonization is shown on the right. (B) Time-lapse microscopy of the filamentation of CB15 cells in spent medium with glucose in a microfluidic device to monitor the process of filament formation. Cells labeled with an asterisk originated from the first cell. Arrowheads indicate a division event during filamentation.

We then decided to look for other potential survival advantages that filamentous cells might have. It is well-established that in their freshwater habitat, C. crescentus cells are able to live either planktonically or in biofilms (7, 8). In most cell biological studies, the wild-type strain NA1000 has been used, which is deficient in production of the adhesive polysaccharide holdfast and biofilm formation and thus has different growth dynamics during surface colonization than would be expected in nature. Hence, we decided to use holdfast-producing wild-type strain CB15 and followed its growth behavior in a flow chamber using a microfluidics device (Fig. 6A) (see Materials and Methods). A few hours after loading of the chamber, individual cells began to attach to the surface, leading to the formation of a cell monolayer. Cell division and continued attachment of planktonic cells led to the formation of a multilayer biofilm within 15 h (Movie S2). Remarkably, following continued incubation in the chamber, we observed that filamentous cells emerged that crossed the biofilm and thus connected the innermost part of the biofilm to its outer surface (Fig. 6A; see also Movie S3). We reasoned that reaching the biofilm surface might allow the cells that are at the bottom of a biofilm to better access nutrients and to release progeny into their surroundings. In support of the latter idea, we observed that cell divisions continued to occur sporadically (Fig. 6B) during cell filamentation and that cells of various sizes budded off from the parent cell filaments while following their formation in spent medium by time-lapse microscopy using microfluidics. Together, these data suggest that filamentous cells are part of C. crescentus biofilms and that their elongated shape might facilitate nutrient uptake and cell dispersal.

10.1128/mBio.01557-19.9 MOVIE S2 Time-lapse movie of initial microfluidic chamber colonization by C. crescentus CB15. Download Movie S2, AVI file, 0.9 MB. Copyright © 2019 Heinrich et al. 2019 Heinrich et al. This content is distributed under the terms of the Creative Commons Attribution 4.0 International license.

10.1128/mBio.01557-19.10 MOVIE S3 Zoom through a four-day-old biofilm grown in a microfluidic chamber, showing filamentous cells that cross the biofilm. Download Movie S3, AVI file, 0.6 MB. Copyright © 2019 Heinrich et al. 2019 Heinrich et al. This content is distributed under the terms of the Creative Commons Attribution 4.0 International license.

## DISCUSSION

Environmentally induced bacterial filamentation is an often-observed but poorly understood phenomenon. Here, we studied the morphological transition of the alphaproteobacterium C. crescentus when incubated for several days in stationary phase, during which cells lose their characteristic swarmer and stalked-cell morphologies and instead gain helical filamentous morphologies. Our data demonstrate that this phenotypic switch is a metabolic response that is triggered by phosphate limitation in combination with alkaline pH and high levels of ammonium. These conditions occur in freshwater lakes as a result of the algal blooms that occur during the warm summer months, suggesting that filamentous growth of C. crescentus is a common response to its natural environment.

### Molecular mechanisms leading to cell filamentation.

Under optimal conditions, bacteria tightly coordinate cell cycle events with growth rate. Timely initiation of DNA replication and cell division ensures that a constant cell size and shape is maintained. Our data highlight that in the filamentous cells isolated from the late stationary phase, the coupling between cell growth and the cell cycle is lost, resulting in cells that continue to grow in the absence of DNA replication and cell division. We found that the block of cell cycle progression is due to the downregulation of major cell cycle regulators, while proteins required for core metabolism and housekeeping remain present. Interestingly, phosphate depletion alone was found to be sufficient to uncouple cell growth from the cell cycle. This is in contrast to other types of starvation, namely, carbon and nitrogen starvation, during which *Caulobacter* arrests the cell cycle while growth ceases, causing cells to maintain their normal size (24, 25). Interestingly, although phosphate-starved cells demonstrate dramatic stalk elongation (Fig. 4E) (26), we did not observe such stalk elongation when phosphate starvation was combined with high pH and high levels of ammonium (Fig. 4F), suggesting that the combination of excess ammonium and high pH somehow restricts stalk elongation.

We attribute the cessation of cell cycle functions mainly to the strongly reduced levels of CtrA and DnaA in the filamentous cells. CtrA and DnaA comprise the two core modules of the *Caulobacter* cell cycle (27, 28). Absence of CtrA results in downregulation of genes encoding important cell cycle regulators (e.g., SciP, CcrM) as well as genes required for morphogenesis and cell division (29). A strong reduction of DnaA prevents DNA replication initiation and affects the expression of around 40 genes, several of which encode proteins involved in cell cycle regulation (e.g., GcrA, MipZ, and FtsZ) (30). Previous work had already revealed that the abundances of DnaA and CtrA are subject to tight environmental control. In the case of DnaA, it was shown that carbon starvation reduces protein translation by a posttranscriptional mechanism and that unfolded protein stress induces fast DnaA degradation by the protease Lon (31, 32). CtrA abundance has been shown to be regulated by environmentally controlled activity of the histidine kinase CckA (12). We consider it likely that similar mechanisms are in place that account for the downregulation of CtrA and DnaA under the conditions described here; however, the exact pathways remain to be elucidated.

While our data explain how cells become filamentous, the reason for their helical cell shape remains less clear. It is well established that specifically blocking cell division, for example, by disrupting CtrA or major divisome components, leads to filamentous cells (18, 33). However, in such cases, cell filamentation is not associated with the helical shape. Similarly, cells depleted for phosphate alone did not show this characteristic cell curvature (Fig. 4E). The helical shape emerged only under conditions of phosphate depletion, alkaline pH, and increased ammonium concentrations. Our proteomics data did not show that CreS was strongly upregulated in these cells. Therefore, we suggest that there is another reason for the helical shape of the filamentous cells under these conditions. The late-stationary-phase filaments were thinner than FtsZ-depleted cells of a similar length (Fig. 1B) and were also thinner than phosphate-starved cells (see Fig. S3C in the supplemental material), neither of which has a helical shape. Thus, it is possible that the decreased width of the late-stationary-phase filaments allows CreS to more strongly influence cell shape, resulting in more pronounced cell bending. It is also possible that the metabolic enzyme CtpS contributes to the helical shape of the late-stationary-phase filamentous cells. CtpS depletion has been shown to increase cell curvature in a CreS-dependent manner (34). In E. coli, CtpS polymerization is induced by its product CTP (35). Although we did not observe appreciable changes in CtpS levels in late-stationary-phase filaments (see Table S1 in the supplemental material), it is possible that the altered metabolic conditions affect cell shape by modulating CtpS filamentation.

### Ecological relevance of C. crescentus cell filamentation.

**FIG 7:**
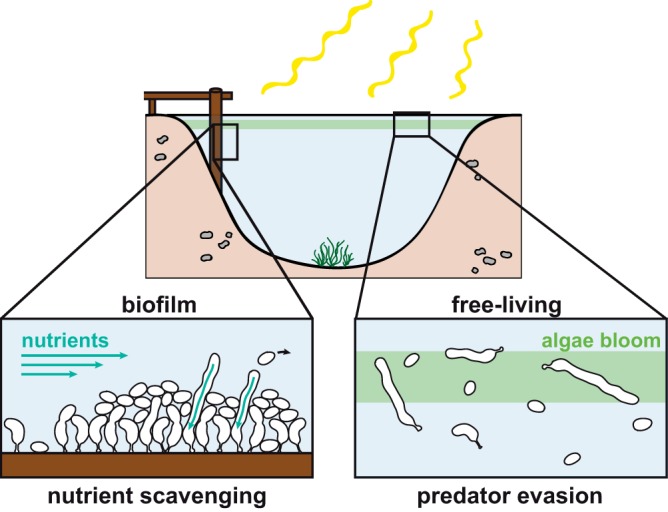
Model of possible occurrences of filamentous C. crescentus cells in the natural habitat. When grown in a biofilm, filamentous cells reach beyond the biofilm surface, which could aid in nutrient scavenging. Filamentation can also be induced below the surface of the water column, where levels of algal bloom and, as a consequence, phosphate concentrations drop and levels of nitrogen and pH increase. Free-living filamentous cells could be protected against predator grazing.

Having seen that *Caulobacter* cells are able to assume a filamentous morphology in the laboratory in response to external conditions and that similar conditions occur in the natural habitat of *Caulobacter*, we considered what advantages, if any, this morphology might confer. Previous work suggested that filamentation might help free-living aquatic bacteria to evade protist predation (36, 37), particularly under phosphate-limiting conditions (38) (Fig. 7). Our new data highlight that a filamentous cell shape could also be beneficial for biofilm-associated C. crescentus cells (Fig. 7). Production of the adhesin holdfast at the tip of its stalk allows C. crescentus to strongly adhere to surfaces and to form layers of cells that can develop into biofilms (7). Our data demonstrate that filamentous cells can participate in such biofilms and reach from the inner part of the biofilm to the surface. We suggest that elongation toward the surface allows cells to access nutrients and to release progeny into the environment. This would be particularly beneficial under conditions of growth in mixed biofilms alongside other microbial species. Whether the helical shape of the filamentous cells contributes to their biofilm-associated functions remains to be elucidated. However, we consider it possible that it may promote penetration through the biofilm. It has previously been suggested that curvature aids mobility through hydrogels in Vibrio cholerae (39), Campylobacter jejuni (40), and Helicobacter pylori (41). Although the studies mentioned above focused on motility through mucus, bacterial biofilms may have similar properties (42).

To conclude, our work sheds new light on environmentally induced morphological variation in bacteria and highlights that bacterial appearance in the natural environment can differ drastically from that observed under standard laboratory conditions. The finding that conditions that cause filamentation occur in freshwater lakes during the summer algal bloom indicates that water chemistry and variations in the microbial composition of lake habitats affect bacterial morphology (43, 44). This suggests that human-induced eutrophication and water warming affect not only microbial composition (45) but also the morphology and physiology of individual cells. More research will be required to address this hypothesis in the future.

## MATERIALS AND METHODS

### Growth conditions.

Caulobacter crescentus wild-type strain NA1000 and its mutant derivates as well as wild-type CB15 (holdfast positive [holdfast^+^]) were routinely grown in PYE (complex medium) or M2G (minimal medium with 0.2% glucose) at 30°C with shaking at 200 rpm. For phosphate starvation experiments, exponential-phase cells precultured in M2G were washed and transferred to M5G medium lacking phosphate (46). For late-stationary-phase cultures, cells were grown for 10 days in PYEX (PYE medium with 0.3% xylose).

Spent medium was prepared by harvesting late-stationary-phase cultures and subsequent filtration of the supernatant through 0.2-μm-pore-size sterile filters. Nutrients were added to the spent medium and water-based media as described in Table S2 in the supplemental material. The pH was monitored during growth and was adjusted if necessary. Except for the stationary-phase experiments, cultures were back-diluted as needed.

10.1128/mBio.01557-19.7 TABLE S2 Media used in this study. Download Table S2, XLSX file, 0.01 MB. Copyright © 2019 Heinrich et al. 2019 Heinrich et al. This content is distributed under the terms of the Creative Commons Attribution 4.0 International license.

### Bacterial strains.

The strains used in this study were Caulobacter crescentus (or *vibrioides*) CB15, CB2, and CB51; Brevundimonas bacteroides CB7 (16); and Caulobacter crescentus NA1000 (CB15N), a CB15 derivate unable to form holdfast (47), FROS reporter strain MT16 (48), Δ*phoB* strain YB720 (49), *P_xyl_-ftsZ* strain YB1585 (33), and KJ931, which was generated in this study.

To construct strain KJ931 (C. crescentus NA1000 *hfa::P_ald_-mNeonGreen*), the *ald* promoter fused to *mNeonGreen* was inserted upstream of *hfaA*, using a strategy similar to one previously described (32). Briefly, Gibson assembly was used to create a pNPTS138 derivative containing, in order, 528 bp of sequence upstream of *hfaA* (−601 to −74 relative to the *hfaA* start codon), the 212 bp immediately upstream of the *ald* start codon, a *mNeonGreen* codon optimized for E. coli (a gift from O. Besharova and V. Sourjik), and another 521 bp of *hfaA* sequence (−73 to +448 relative to the *hfaA* start codon). Two-step homologous recombination with this plasmid was used to integrate *hfa::P_ald_-mNeonGreen* into C. crescentus NA1000 as previously described (32).

### Microscopy.

For living-cell analysis and time-lapse microscopy, cells were transferred onto a PYE 1% agarose pad with supplementation as required. Otherwise, cells were fixed with 1% formaldehyde and mounted onto 1% agarose pads. Phase-contrast and fluorescence images were taken using a Ti eclipse inverted research microscope (Nikon) with a 100×/1.45 numerical aperture (NA) objective (Nikon). Fiji (ImageJ) was used for image processing. Cells were classified as filamentous if they were more than twice the average length of exponentially growing C. crescentus cells.

Cells were measured using MicrobeJ 5.13l (15) beta (50). Cells were detected using the “Default” setting for thresholding and the “Filament” setting for detection of bacteria. Cell detection was manually corrected where necessary. To determine the cell width, the median width for each cell was used.

### Purification of filamentous cells from late-stationary-phase cultures.

Late-stationary-phase cultures (10 days in PYEX) were mixed with 40% Percoll (GE Healthcare) and centrifuged for 20 min either in 1.5-ml reaction tubes at 10,000 rpm or in 15-ml Falcon tubes at 7,500 rpm in a fixed-angle rotor. Cells from the lower band (filamentous cells) were isolated and washed before being resuspended in the appropriate medium. The top band mostly consisted of small and dead cells.

### Photobleaching.

Strain KJ931 expressing mNeonGreen was incubated for 10 days in PYEX, purified as described above, and mounted on 1% agarose pads. Images were recorded using an LSM 780 confocal laser scanning microscope (Zeiss) with an Argon laser at 488 nm. Bleaching was performed over a 2.7-μm-by-2.7-μm square. A total of 50 cycles of bleaching were performed, followed by a pause of several seconds to allow the fluorescent protein to diffuse throughout the cell. This process was repeated 3 times.

### Flow cytometry.

Samples were prepared as described earlier (32) and analyzed using a BD LSRFortessa flow cytometer. Data were collected for at least 30,000 cells. Flow cytometry data were analyzed with FlowJo.

### Proteomics.

Samples for quantitative proteomics were prepared and processed as previously described (51). Briefly, samples were harvested by centrifugation (for late-stationary-stage filamentous cells after purification as described above) and pellets were washed with water and snap-frozen in liquid nitrogen. Samples were processed in biological duplicate. Protein digestion, TMT10plex isobaric labeling, and mass spectrometry were performed at the Clinical Proteomics Mass Spectrometry facility, Karolinska Institute/Karolinska University Hospital/Science for Life Laboratory. Proteins for which fewer than 3 peptides were detected were discarded. To calculate the average log fold change for different categories of proteins, replicate values were averaged and proteins classified based on entries in the KEGG Orthology database (52), followed by manual changes (Table S1).

### Immunoblotting.

Pelleted cells were resuspended in 1× SDS sample buffer, normalized to the optical density of the culture, and heated to 95°C for 10 min. Protein extracts of cell lysates were then subjected to SDS-PAGE for 90 min at 130 V at room temperature on 12% Tris-glycine gels and transferred to nitrocellulose membranes. To verify equal loading, total protein was visualized using the 2,2,2-Trichloroethanol (TCE) in-gel method (53) prior to blotting. Proteins were detected using the following primary antibodies and dilutions: anti-CtrA (kindly provided by M. Laub) (1:5,000), anti-CcrM (54) (1:5,000), anti-DnaA (28) (1:5,000), anti-DnaK (51) (1:5,000), anti-EF-Tu (clone 900, Hycult catalog no. HM6010) (1:2,000), anti-FtsZ (55) (1:10,000), anti-GcrA (56) (1:4,000), anti-GroEL (57) (1:10,000), anti-Lon (kindly provided by R.T. Sauer) (1:10,000), anti-RpoB (clone 8RB13, Biolegend catalog no. 663903) (1:2,000), and anti-RpoD (clone 2G10, BioLegend catalog no. 663203) (1:2,000). A 1:5,000 dilution of secondary horseradish peroxidase (HRP)-conjugated antibody (Thermo Scientific) and SuperSignal Femto West reagent (Thermo Scientific) were used for detection. Blots were scanned with a ChemiDoc (Bio-Rad) system. Images were processed with Bio-Rad Image Lab, Adobe Photoshop, and ImageJ, and the relative band intensities were quantified with Image Lab.

### Live/Dead stain.

Viability of cells was analyzed with the LIVE/DEAD BacLight fluorescence stain from Life Technologies (Thermo Fisher Scientific). The two dye components were combined at a 2:1 ratio of component A with component B. A 1-μl volume of the mixed dye was added to 300 μl of liquid culture from 0-, 0.5-, 1-, 2-, 4-, 7-, and 10-day-old PYEX cultures, and the mixture was incubated for 30 min at room temperature (RT). Stationary cultures were diluted 1:5 in PYE medium before imaging was performed.

### Environmental database.

Water chemistry data of Lake Ekoln were extracted from Miljödata-MVM (2018; Swedish University of Agricultural Sciences [SLU], national data host for lakes and watercourses, and national data host for agricultural land, http://miljodata.slu.se/mvm/ [accession date, 7 March 2018]).

### Bacterioplankton community analyses.

Water samples from Lake Erken (59.84N, 18.64E) were collected as integrated samples from the entire water column during periods of mixing and from the upper epilimnion during periods of thermal stratification across the period 2011 to 2015. Samples were collected weekly or monthly, and the seasonal coverage was as follows: August to December 2011, January to November 2012, January to August 2013, June to December 2014, and February to April 2015. The methodology used for sample preparation, sequencing, and sequence analysis was described previously by Sinclair et al. (58). Briefly, DNA from bacterial cells captured on 0.2-μm-pore-size polyethersulfone membranes was extracted by the use of a MoBio Power Soil DNA extraction kit. The variable V3 and V4 regions of bacterial 16S rRNA genes were PCR amplified for 20 cycles with bacterial primers 341F (CCTACGGGNGGCWGCAG [59]) and 805R (GACTACHVGGGTATCTAATCC [60]) featuring Illumina adapter sequences at the 5′ ends. In a second PCR targeting these adapter sequences, amplicons from individual samples were individually barcoded and equipped with standard Illumina handles prior to Illumina MiSeq sequencing with 2 × 300-bp chemistry. Sequences have been deposited in the European Nucleotide Archive (ENA) (see below). USEARCH was then used to cluster sequences into operational taxonomic units at 97% identity, and the sequences were taxonomically annotated by alignment to the Ribosomal Database Project (RDP v. 16 [61]).

### Water chemistry analysis.

Nutrients and pH were provided by the certified water chemistry laboratory of the Erken Laboratory using methods developed and maintained by the Swedish and International Standards Organization (SS-ISO). pH was measured with a pH meter according to SS 02 81 22 (2), PO_4_-P was analyzed as molybdate reactive phosphorus according to standard SS EN ISO 6878:2005, and NH_4_-N was analyzed colorimetrically with flow injection analysis according to standard SS EN ISO 11732:2005.

### Fluorescent *in situ* hybridization (FISH).

A specific FISH probe was designed based on the Caulobacteraceae probe (accession no. pB-377); however, three nucleotides were modified in order to obtain 100% sequence identity with the Caulobacter crescentus 16S rRNA sequence, resulting in oligonucleotide 5′-TTCCACATACCTCTTCCG-3′. The probe was labeled with Atto 488 at the 5′ and 3′ ends (Biomers, Germany).

Environmental samples were prefiltered with a filter net (pore size, 100 μm). The fluorescent *in situ* hybridization (FISH) protocol was adapted from Glöckner et al. (62). Samples were fixed prior to filtration with 2% paraformaldehyde. Cells were then filtered on white polycarbonate filters (0.4-μm pore size, 25-mm diameter) supported by Whatman glass microfiber filters (grade GF/F; 0.7-μm pore size, 25-mm diameter) by applying vacuum. Filters were air-dried, cut in 8 sections, and stored at –20°C. All of the following steps were conducted in the absence of light. One filter section per sample was incubated in 180 μl hybridization buffer containing 0.9 M NaCl, 20 mM Tris-HCl (pH 8), 40% formamide, and 0.01% (vol/vol) SDS plus 20 μl probe containing 50 ng oligonucleotide at 46°C for 2 h. The filters were transferred to 50 ml prewarmed (48°C) washing buffer containing 5 mM EDTA (pH 8), 20 mM Tris-HCl (pH 8), 46 mM NaCl, and 0.01% (vol/vol) SDS and incubated at 48°C for 15 min. The filters were then transferred to double-distilled water (ddH_2_O) for 5 min, to 70% ethanol for 1 min, and finally to 96% ethanol for 1 min before being subjected to air-drying. DAPI staining solution (final concentration, 2 μg/ml) was mixed with ProLong Glass Antifade Mountant (Invitrogen), and a 50-μl volume was spotted onto a filter section on a glass slide before being covered with a cover slip. Samples were incubated for at least 15 min at RT prior to microscopy. Fluorescence microscopy was conducted as described above using DAPI, yellow fluorescent protein (YFP), and TxRed filters. Cells that fluoresced in the TxRed channel were omitted from the analysis, since this was indicative of the autofluorescence that would often also be present in the YFP channel.

### Microfluidics.

CB15 (holdfast^+^) cells were grown to the exponential phase in PYE medium and then loaded into a microfluidic plate (B04A-03; Merck Millipore). Medium flow and temperature were monitored with a CellASIC ONIX2 microfluidic system (Merck Millipore). The chamber was heated to 30°C, and the flow pressure was set at 2 lb/in^2^. When the chamber was not attached to the system, it was incubated at 200 rpm and 30°C on a shaker in an airtight bag.

The growth chamber itself was constructed to trap cells via the use of a loading-pressure-dependent flexible ceiling. However, our intention was to follow *Caulobacter* surface colonization without the limitations imposed by the restrictions of the chamber. Therefore, we imaged cells in the broader and wider medium channels that lead into the growth chamber. Under those conditions, *Caulobacter* swarmer cells can swim along the channels and have space to settle on surfaces without getting trapped by the ceiling. After surface colonization, the channels were regularly flushed with diluted rich medium applied at 2 lb/in^2^ to keep the channel above the biofilm mostly cell free.

### Survival assay.

C. crescentus cultures were cultured in PYEX. Exponential-phase cultures were grown to an optical density at 600 nm (OD_600_) of below 0.4. Stationary-phase cultures were obtained from overnight cultures with an OD_600_ of 1.2 to 1.5. Filamentous cells were obtained from 10-day-old cultures by gradient centrifugation with Percoll as previously described. For the sucrose treatment, the OD_600_ was adjusted to 0.033 in medium containing 1 M sucrose. After 1 h of incubation at 30°C with shaking, cells were serially diluted in PYE medium and plated to determine CFU counts. For the acid stress conditions, the OD_600_ was adjusted to 0.15 in medium that had been preadjusted to pH 4.5 with HCl and the cells were incubated for 1 h before plating. For the oxidative stress conditions, the OD_600_ was adjusted to 0.15 in medium containing 0.25 mM hydrogen peroxide and the cells were incubated for 1 h before plating. For the heat stress conditions, the OD_600_ was adjusted to 0.15 and the cultures moved to 50°C for 4 min before plating. Serial dilutions were made, and the cells were plated on PYE medium and incubated for 2 days at 30°C. Plates with reasonable numbers of colonies (30 to 300) per plate were counted, and CFU counts per milliliter were calculated and normalized to the OD_600_ of the sample plated.

### Data availability.

Sequences have been deposited in the European Nucleotide Archive (ENA) under accession number PRJEB20109.
