# Supplementary figures and images for: Molecular Basis and Ecological Relevance of Caulobacter Cell Filamentation in Freshwater Habitats
Source: mBio. 2019 Aug 20;10(4):e01557-19. doi: 10.1128/mBio.01557-19 (PMC6703425; doi:10.1128/mBio.01557-19)

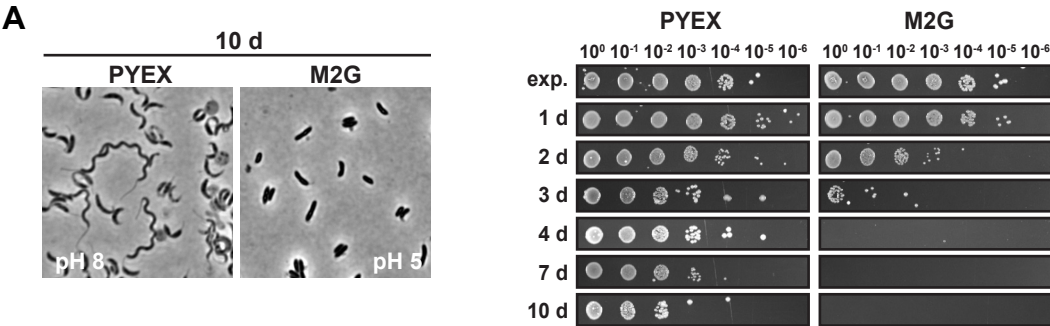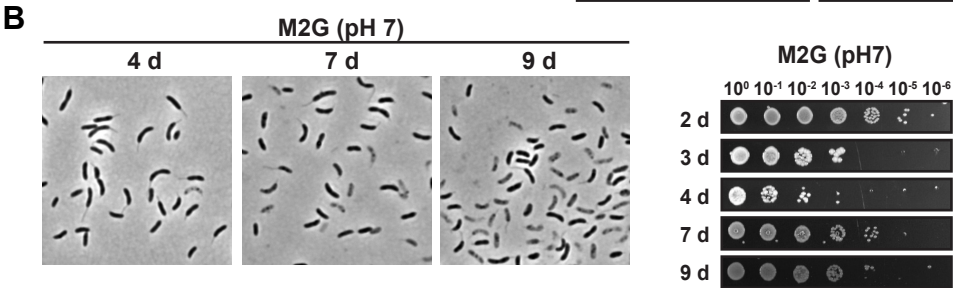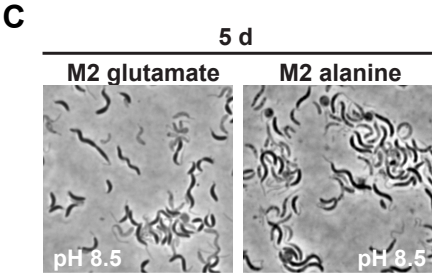

Supplement: FIG S1 [file mBio.01557-19-sf001.pdf]

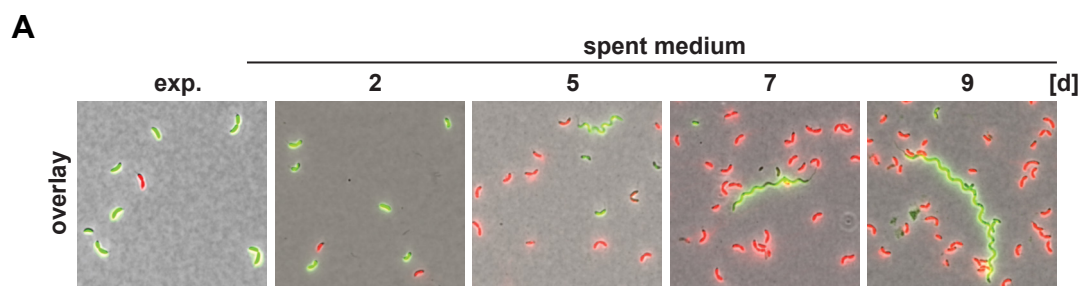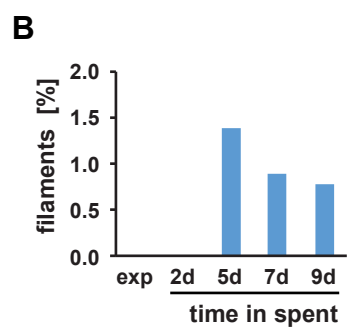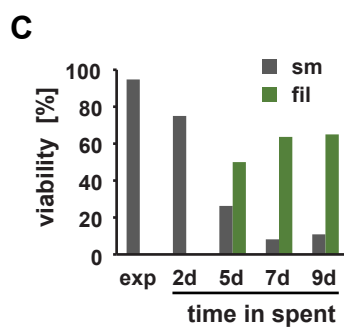

Supplement: FIG S2 [file mBio.01557-19-sf002.pdf]

**A**

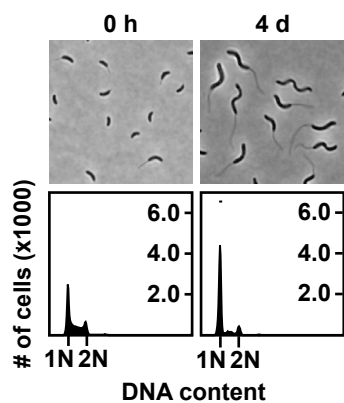

**B**

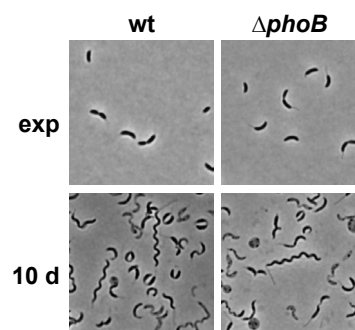

**C**

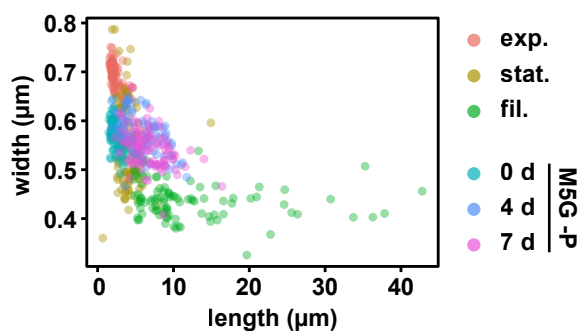

**D**

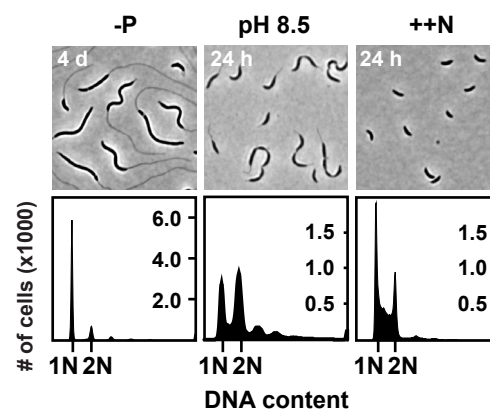

**E**

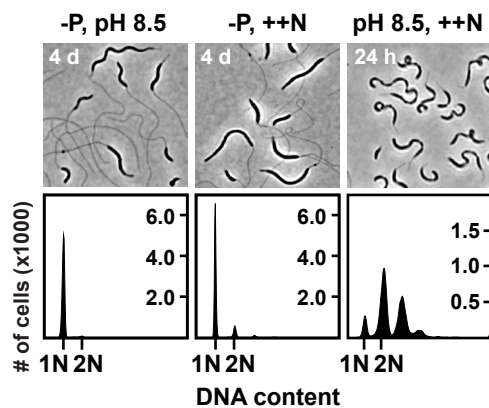

**F**

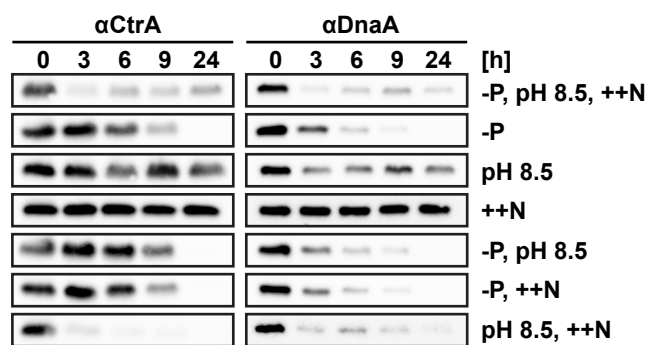

**G**

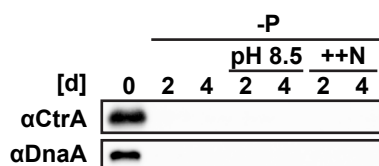

Supplement: FIG S3 [file mBio.01557-19-sf003.pdf]

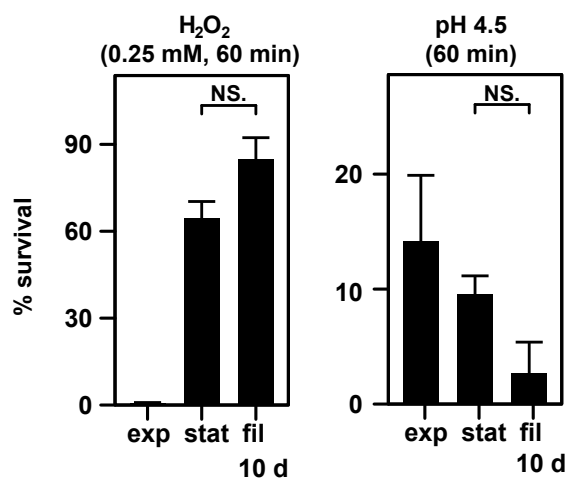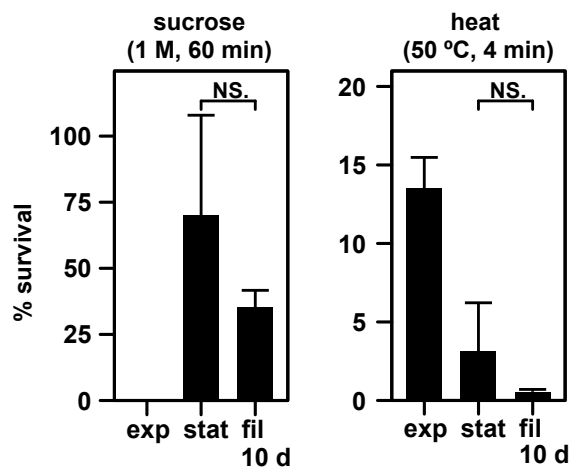

Supplement: FIG S5 [file mBio.01557-19-sf005.pdf]
