# Supplementary material for: Molecular Basis and Ecological Relevance of Caulobacter Cell Filamentation in Freshwater Habitats
Source: mBio. 2019 Aug 20;10(4):e01557-19. doi: 10.1128/mBio.01557-19 (PMC6703425; doi:10.1128/mBio.01557-19)
Supplement: FIG S4 [file mBio.01557-19-sf004.pdf]

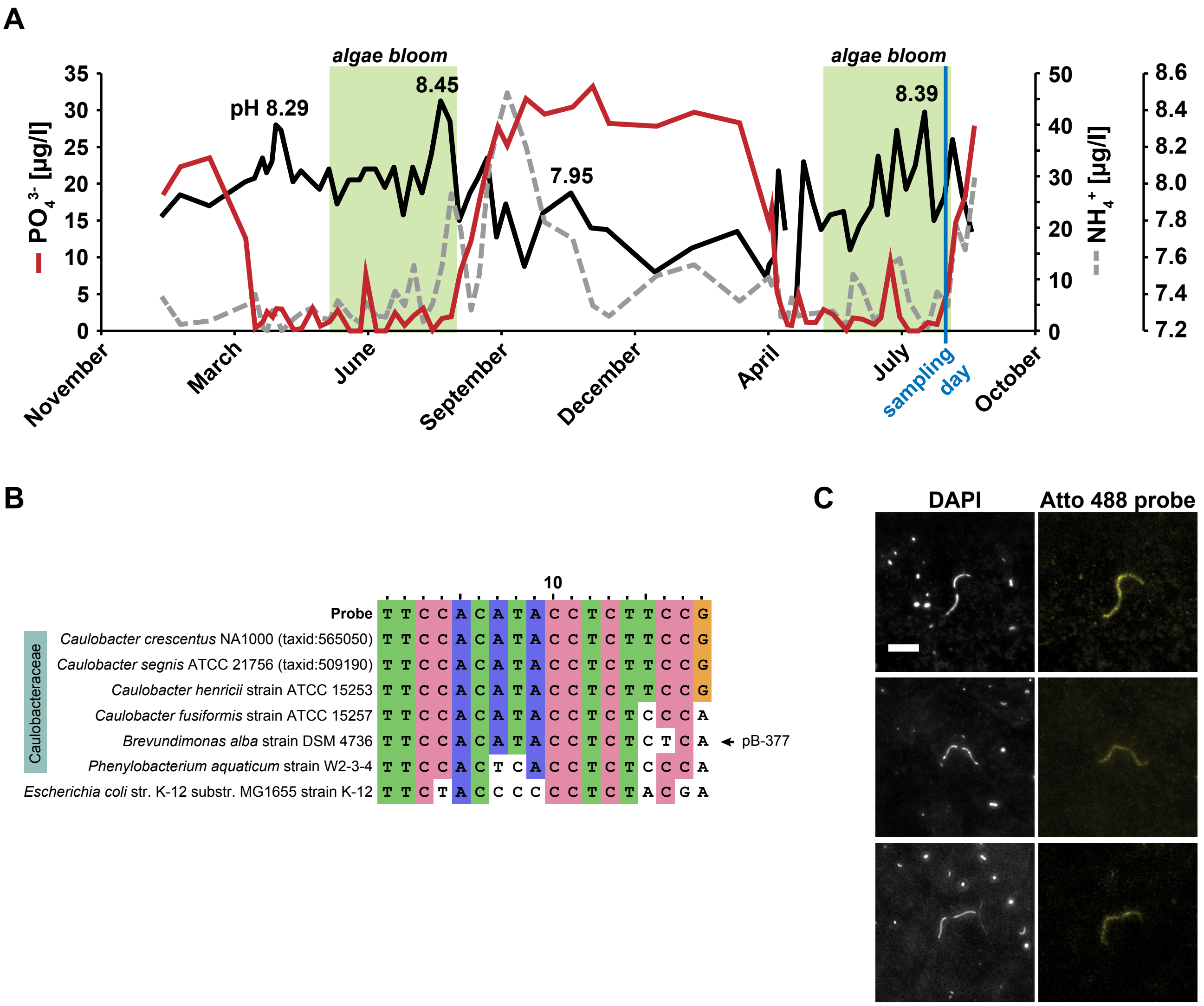
A
algae bloom
algae bloom
pH 8.29
8.45
7.95
8.39
PO4 3- [µg/l]
NH4 + [µg/l]
pH
35
30
25
20
15
10
5
0
50
40
30
20
10
0
8.6
8.4
8.2
8.0
7.8
7.6
7.4
7.2
November
March
June
September
December
April
July
sampling day
October
B
10
Probe T T C C A C A T A C C T C T T C C G
Caulobacter crescentus NA1000 (taxid:565050) T T C C A C A T A C C T C T T C C G
Caulobacter segnis ATCC 21756 (taxid:509190) T T C C A C A T A C C T C T T C C G
Caulobacter henricii strain ATCC 15253 T T C C A C A T A C C T C T T C C G
Caulobacter fusiformis strain ATCC 15257 T T C C A C A T A C C T C T C C C A
Brevundimonas alba strain DSM 4736 T T C C A C A T A C C T C T C T C A
Phenylobacterium aquaticum strain W2-3-4 T T C C A C T C A C C T C T C C C A
Escherichia coli str. K-12 substr. MG1655 strain K-12 T T C T A C C C C C C T C T A C G A
Caulobacteraceae
pB-377
C
DAPI
Atto 488 probe
